# Buccal Fat Pad Excision for Facial Rejuvenation: The Relationship Between the Resected Position and Its Influence on Facial Shape and Volume

**DOI:** 10.1093/asjof/ojad089

**Published:** 2023-10-04

**Authors:** Takayuki Kubo

## Abstract

**Background:**

To date, facelift surgery has been the most common choice for those seeking antiaging solutions. However, buccal fat pad (BFP) excision has also been utilized recently.

**Objectives:**

An interrelation between the BFP, resection area, and its influence on facial shape and volume is scrutinized to achieve “tailor-made” outcomes in patients with BFP-related symptoms.

**Methods:**

Patients were categorized into 2 groups: Type I with a bottom-heavy face and Type II with ptotic lower cheeks, typically seen in older people. The lower face was divided into upper and lower segments bilaterally. Then, the relationship between the resected position of the BFP and its influence on facial shape and volume was studied.

**Results:**

BFP excision was performed for 133 patients (118 females and15 males) between May 2020 and June 2022. Sixty-one of these patients were categorized into Type I (39 patients) and Type II (22 patients) and were followed up for 12 months postoperatively. The volume of all lower facial segments decreased postoperatively in both types of patients. The variation rate of the upper segment volume in Type II patients was less than that in Type I patients. The results were consistent with a technique that did not remove large chunks of the BFP from the upper segment in Type II patients.

**Conclusions:**

BFP excision is an effective facial rejuvenation treatment if proper candidates are selected. To improve the accuracy of BFP excision, the relationship between the resected position of the BFP and its influence on facial shape and volume should be well understood.

**Level of Evidence: 3:**

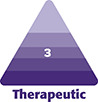

The buccal fat pad (BFP) was first reported in the year 1732 and its histology was examined by Xvier Bichat in 1802.^1^ Although the precise role of the BFP and its function remained unclear for a long time, some previous papers suggested that the BFP is a spacer between the buccinator (suckling) and the masseter (chewing),^2,3^ and could also act as a cushion to avoid facial nerve injuries from external force.^4^ Traditionally, BFP excision was performed to treat lip lacerations or traumatic facial defects using the BFP as a pedicle frame to repair any defect in reconstructive surgery.^5-7^ During infancy, the appearance of the BFP is significant in making the face look chubby, and it remains large until mandibles develop to cover the BFP.^8^ During growth, the BFP typically becomes less prominent, but faces with a large BFP often demonstrate typical fan-shaped cheeks, the so-called chipmunk face. BFP excision has been the permanent remedy for the copious face shape, producing successful results, as such a face does not regenerate after resection. If a large BFP remains untreated, it can descend with age and contribute to a sagging jawline. As such, BFP excision has become a popular method to prevent the associated sagging of an aging face.

The face is composed of 3 elements: skin, soft tissues, and bone. The facial surface is separated into several layers, including skin, subcutaneous tissue, musculoaponeurotic layer, areolar tissue, and periosteum.^9^ Thin fat layers called the suborbicularis oculi fat (soof),^10^ malar, and jowl fat are also included in the subcutaneous tissue. The face differs from the rest of the body in that there are 2 deep fat pads, 1 (periorbital fat pads) located at the periorbital region and the other (BFP) at the masticatory space in the cheeks.^11^ Commonly, early aging changes occur in the periorbital region because of its delicate and intricate structure. For instance, lower eyelid fat pads are supported superiorly by the orbicularis muscle and its retaining ligaments and inferiorly by the zygomatic retaining ligaments. Attenuation of the retaining ligaments and supporting tissues around the lower eyelids occurs gradually with aging. When ligament laxity reaches the point where it can no longer support the lower eyelid fat pads, the pads can burst out anteriorly, and this phenomenon is commonly referred to as “eyebags.” Surgery to counter this condition is well established.^12^ The same theory applies to the BFP in the cheeks, as it shifts downward with aging because of a weakening of the retaining ligaments attached to the surrounding tissues. The BFP located deep inside the cheeks comprises 4 parts: body, extension, pterygoid, and temporal in the masticator space. The BFP is important in maintaining cheek fullness, and it is supported by the retaining ligaments attaching to the masticators and zygomatic bone.^13^ If the BFP is large and heavy, its extension (mainly distal) part descends downward where the premasseteric space located at the lower portion of the masseter plays an important role in the formation of jowls.^14^ When the mandibular ligaments are attenuated, the premasseteric space occasionally becomes loose or dilates enough to have the distal part of the BFP extension fall into its space. If droopiness at the jowls becomes more conspicuous, the BFP could be the main cause of ptotic jawlines, and this phenomenon is called “BFP pseudo-herniation.”^15^

Although facelift surgery can deliver safe and effective results and facial rejuvenation techniques continue to evolve remarkably, nonsurgical methods such as facial fillers, thread lifting, skin-tightening machines with radiofrequency (RF), and ultrasound energy are becoming and have become popular because of their minimal invasiveness. However, the effects of each therapy are minor when compared with surgery. A simple surgery such as BFP excision, which merely reduces the weight and volume burden in the lower cheeks, can be regarded as another ancillary surgical option, and it has been showing good results in the treatment of aging faces. Hence, this study examines the true value of BFP excision in terms of the relationship between the resected position of BFP and its influence on facial shape and volume.

## METHODS

**Figure 1.**
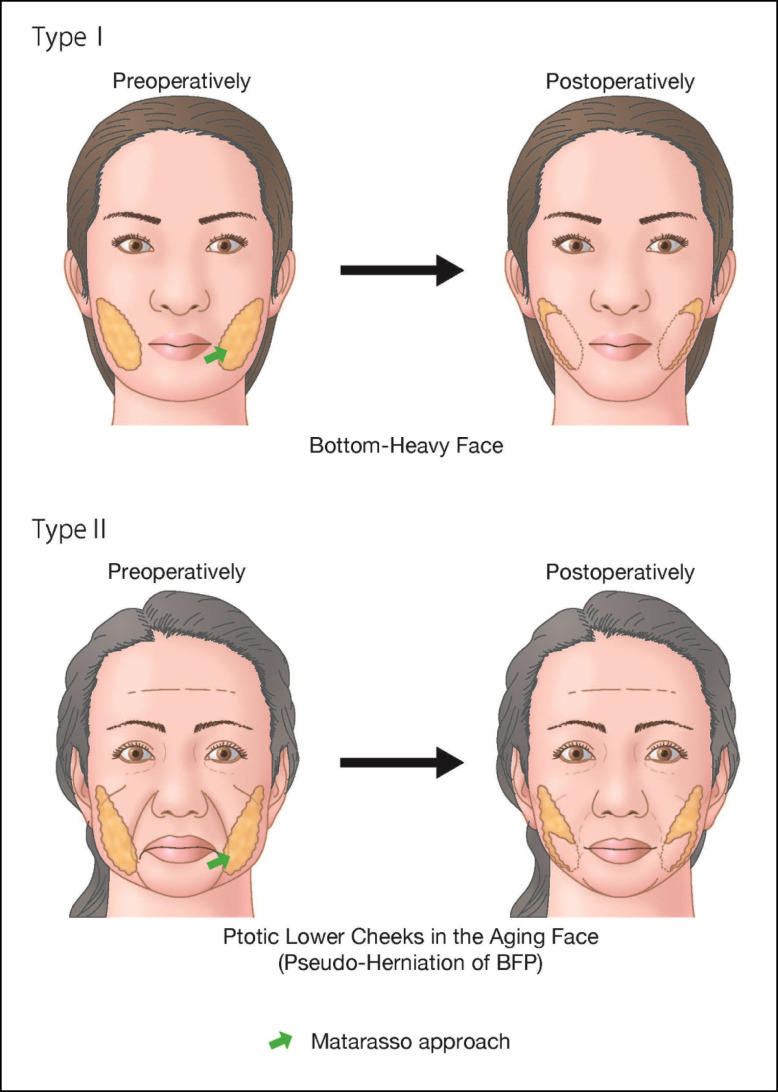
The relationship between the resected position of the buccal fat pad (BFP) and its influence on the facial shape and volume. Type I is a bottom-heavy face commonly seen in the northern Asian population. Type II is a typical aging face with a ptotic lower cheek sometimes accompanied by pseudoherniated BFP. The Matarasso (an arrow) approach is used for BFP excision in both Type I and II patients, starting with a resection of the distal part of BFP extension. The resected position and range of the BFP is differentiated in each type. The BFP is illustrated as a yellow mass inside the cheeks.

**Figure 2.**
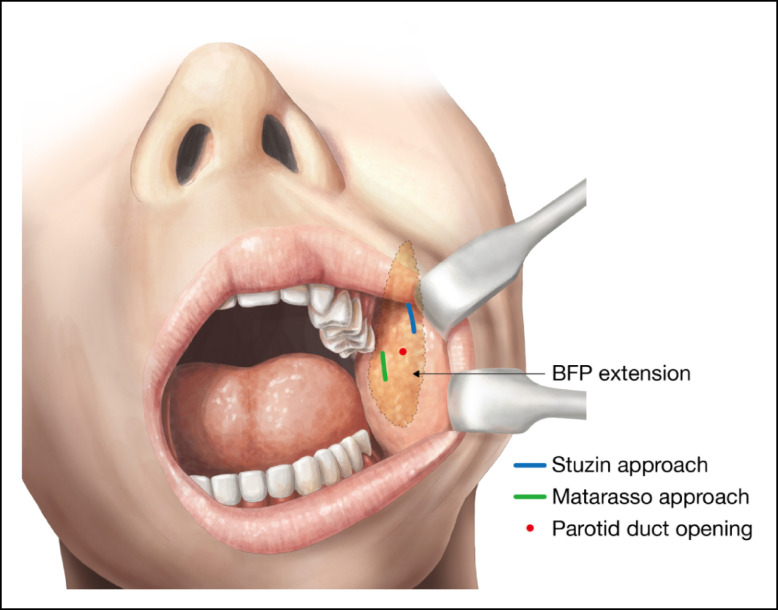
The Stuzin approach (a line above the parotid duct): A small longitudinal incision is made at the maxillary gingivobuccal sulcus above the parotid ducts, directly accessing the buccal fat pad (BFP) pocket. The Matarasso approach (a line below the parotid grand): A small incision is made at the oral vestibule approximately 1 cm below the parotid duct exit. Both the oral mucosa and the buccinator need to be dissected to reach the septum enveloping the BFP. The red circle is the parotid gland exit. The shaded yellow oval is the BFP extension.

This study follows the Declaration of Helsinki and its guiding principles. BFP excision was carried out on 133 patients (118 females and 15 males) who visited our clinic for the treatment of copious or ptotic lower cheeks between May 2020 and June 2022. Sixty-one patients (59 females and 2 males) were reviewed up to 12 months postoperatively, and they were examined for an analysis of the interrelation between the resected position of the BFP and its influence on the facial volume and shape. BFP excision was performed only in patients with objective BFP protrusion or in those with ptotic cheeks of the anterior-medial portion of the face. All candidates had a northern Asian background, where face volume reduction can be more beneficial than skin tightening through a facelift. Patients predisposed to severe bleeding were contraindicated from this surgery, and for patients prescribed antithrombotic drugs, intake was withdrawn 5 days prior to the day of surgery. Informed consent was received, and all associated risks were explained prior to the surgery. One surgeon performed the whole process, including the 12-month postoperative follow-up. Facial shapes of the patients were tentatively grouped into 2 types, as shown in Figure 1. Patients with a heavy and large face or bottom-heavy face typically seen in the northern Asian population were categorized as Type I. Aging patients with ptotic lower cheeks (jawlines), occasionally accompanied by pseudoherniation of the BFP, were categorized as Type II. Then, 39 and 22 patients were diagnosed as Types I and II, respectively, by the author. There are 2 surgical approaches in BFP excision, as shown in Figure 2. One is called the Stuzin approach, in which the incision is made at the maxillary gingivobuccal sulcus above the parotid ducts, directly accessing the BFP pocket, succeeding to the proximal part of the BFP extension. The other is the Matarasso approach, entering from the oral vestibule approximately 1 cm below the parotid duct exit, then dissecting the oral mucosa and subsequently the buccinator to the distal part of the BFP extension. The Matarasso approach was used for BFP excision in both patient types. Most of the BFP extensions were resected for the Type I patients, so that the whole lower cheek was reduced evenly, while only the distal part of the BFP extension, which caused aging jawlines or pseudoherniation, was resected for Type II patients, as shown in Figure 1.

**Figure 3.**
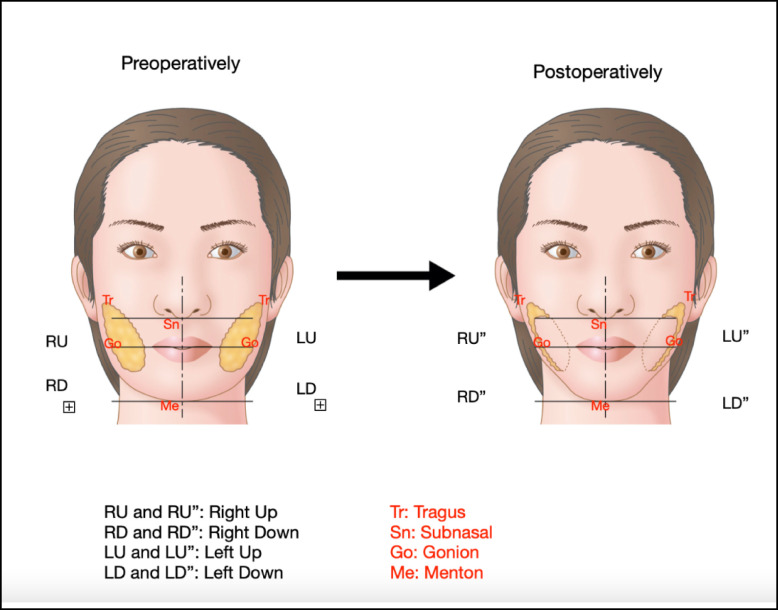
The lower face is split into 4 parts for the volumetric analysis. The bilateral lower face is divided into upper and lower segments by 3 horizontal lines drawn on the face. The upper horizontal line is drawn just below the nasal tip designated as the subnasal (Sn) of a facial landmark extending to the point below the tragus (Tr). The middle horizontal line is drawn on the center of the lips extending to the bilateral gonion (Go). The bottom line is drawn on the center of the menton (Me). A major part of the BFP extension is resected evenly from Type I face in this illustration. The divided lower facial segment is designated as right up (RU), right down (RD), left up (LU), and left down (LD) preoperatively and RU″, RD″, LU″, and LD″ postoperatively.

**Figure 4.**
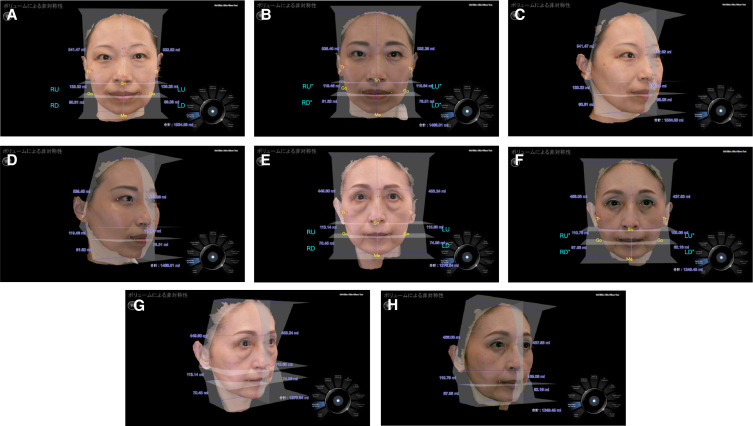
Each segment of the preoperative and 12-month postoperative lower facial volume was measured by using a 3-dimensional (3D) photograph analysis. (A, C, E, G) Preoperative and (B, D, F, H) 12-month postoperative 3D photographs (front and right oblique) of a typical patient in (A-D) Type I and (E-H) Type II were presented with the volume of each facial segment. The patients in Types I and II were a 48-year-old female and a 62-year-old female, respectively.

The lower face was split for analyzing the interrelation between the resected position of the BFP and its influence on the facial shape and volume, as shown in Figure 3. The bilateral side of the lower face was divided into upper and lower segments by 3 horizontal lines drawn on the skin. The upper horizontal line was drawn just below the nasal tip, designated as subnasal (Sn), with a facial landmark extending to the point below the tragus (Tr). The middle horizontal line was drawn on the center of the lips extending to the bilateral gonion (Go). The bottom line was drawn on the center of the Mentum as menton (Me). Facial volumes of 4 lower facial segments divided by those lines were designated as right up (RU), right down (RD), left up (LU), and left down (LD) preoperatively and RU, RD, LU, and LD postoperatively. The Morpheus 3D software (Morpheus Co., Ltd, Yongin, Gyeonggi, South Korea) was used to analyze the volume difference of the upper and lower segments of the bilateral lower face by comparing preoperative and 12-month postoperative 3-dimensional (3D) pictures, as shown in Figure 4. Measurement and analysis were performed by the author using the Morpheus software by requisitioning the services of a technician from Morpheus Co., Ltd.

Patients in this study were demographically analyzed, and the excised bilateral BFP weight difference of all 113 patients was statistically compared using the Mann–Whitney test. The validity of the resected BFP position and its influence on facial volume modification was statistically evaluated by comparing the preoperative and 12-month postoperative volume differences of each lower facial segment in 61 follow-up patients. Those differences were statistically analyzed using the paired *t* test. Preoperative and postoperative volumetric discrepancies of each lower facial segment in both Types I and II were presented as the variation rate (VR). The VR was calculated by using the next formula; for example, the VR of the right upper segment (RU) = (RU″ − RU)/RU × 100. Then, the VR of each lower facial segment (RU, LU, RD, and LD) between Types I and II was statistically compared using the unpaired *t* test. Concomitant surgeries, satisfaction rate, and complications were also elucidated. One case of BFP excision, which was followed by a subsequent minimally invasive facelift performed 18 months after the initial surgery, is presented in the form of 12-month postoperative photographs.

### Surgical Technique

#### BFP Excision

Mild sedation using Diazepam (10-20 mg) and hydroxyzine hydrochloride (12.5-25 mg) was administered prior to the surgery (Video 1). The patient was locally anesthetized with an injection of approximately 6 mL of 1% xylocaine with 1:100,000 epinephrine into each oral vestibule. Precise surgical techniques of BFP excision are omitted here, with published details available in the author's previous paper.^16^ For a Type I face, the BFP was resected using the Matarasso approach, starting at the distal part of the BFP extension, gradually proceeding up to the proximal part, and leaving the top and outer margins of the BFP to allow smooth cheek contouring. For a Type II face, only the distal part of the BFP extension was resected using the Matarasso approach to improve jawlines and pseudoherniation of the BFP, and the proximal part of the BFP extension was preserved to avoid a gaunt appearance. The surgical approach and method for each type is shown in Figure 1. After these procedures, the wound was closed with a 6-0 nylon suture, and it was removed at the postoperative 7 days. The patient was kept in a reclining supine position, and the face was treated with ice packs and a compression garment for at least 1 h postoperatively.

#### Minimally Invasive Facelift

One patient was followed up with a mini facelift 18 months after the initial BFP excision (Video 2). The mini facelift technique is described as follows: An incision line was made along the tragus, extending superiorly 45° bending to the vertical inside the sideburns. The range of subcutaneous dissection was also designed on the facial surface. The distal point of the oval dissection area was two-thirds of the distance to the lateral mouth corner. After the patient was sedated intravenously, local anesthesia (1% xylocaine with 1:100,000 epinephrine) was injected into the incision lines, and tumescence fluid (0.25% with 1:400,000 epinephrine) was infiltrated in the designed area. Subcutaneous dissection was carried out using a 2 mm liposuction cannula with vacuum pressure. Liposuction was simultaneously carried out in this area, extending further down to reduce the superficial jowl fat as well. The lateral portion of the zygomatic ligament was bluntly dissected during this procedure. Then, a skin incision was made following the designed line, and dissection continued with blunt end scissors to detach the whole skin from the subcutaneous plane within the designed oval area. A meticulous hemostasis was made each time bleeding was noticed during dissection.

**Figure 5.**
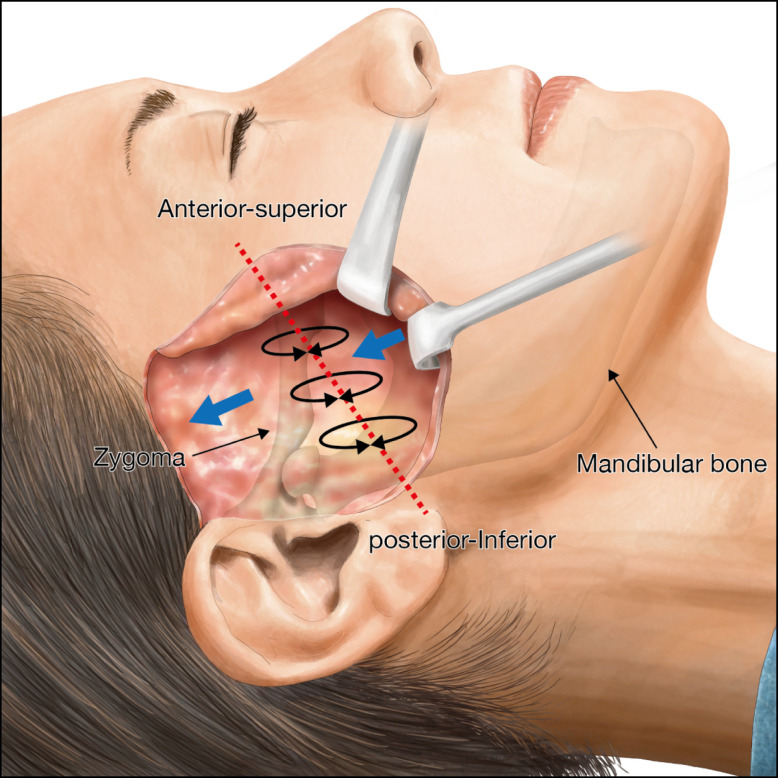
Subcutaneous dissection and the superficial musculoaponeurotic system (SMAS) plication. After the subcutaneous dissection, the skin flap is elevated to expose the SMAS. The SMAS plication is made using a 3-0 polydioxanone (PDS) string at 3 points (the circles) with trisect intervals over the zygoma. A width of approximately 2 to 3 cm of SMAS is plicated in a superior-oblique direction indicated by the arrows.

After sufficient dissection, full mobility of the skin flap detached from the subcutaneous plane was confirmed without any bleeding. Then, the superficial musculoaponeurotic system (SMAS) plication was made using a 3-0 polydioxanone (PDS) string at 3 points with a trisect interval on the midface near the zygoma from anterior-superiorly to posterior-inferiorly, as shown in Figure 5. Each plication suture was made in order to pull the SMAS in the superior-oblique direction, aiming for approximately 2 to 3 cm SMAS shortening, which was anchored to the fascia near the zygoma. After this SMAS plication, the skin flap was draped back over the SMAS in a superior-oblique direction. Excess parts of the skin flap were trimmed off without too much tension when the skin flap was pulled for closure in such a way as to facilitate scarless healing. Subcutaneous sutures and skin sutures were made using 5-0 PDS and 6-0 nylon strings, respectively. Icing and compression were placed for at least 2 h postoperatively, and the wound was reconfirmed to be safe before the patient was discharged.

## RESULTS

**Figure 6.**
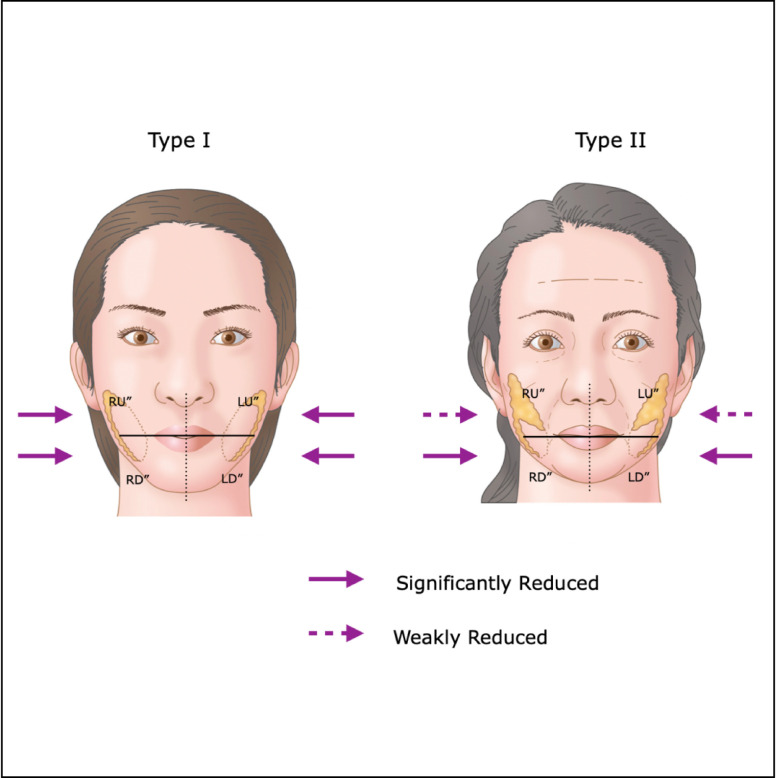
Postoperative volume of the lower face is evenly decreased in Type I shown as 2 purple arrows. However, the postoperative volume decrease of the upper segment of the lower face in Type II is less than that of Type I, which is indicated as purple dotted arrows. This is due to the fact that the proximal part of the BFP extension is resected conservatively in Type II to avoid the postoperative gaunt appearance.

BFP excision was performed for 133 patients (118 females and 15 males) between May 2020 and June 2022. The average age was 47.65 ± 11.99 years (21-79). Extirpated BFP weights were 1.69 ± 0.74 g (0-3.6) for the right and 1.58 ± 0.77 g (0-4.1) for the left. Differences between each side of the extirpated BFP weight were evaluated as not statistically significant (*P* = 2.0) using the Mann–Whitney test. Subsequently, 64 patients (59 females and 5 males) applied for the follow-up postoperatively, and the average follow-up time was 12 months from May 2020 to June 2023. However, 3 patients were excluded from the study because 1 patient was found to have a previous BFP excision and the other 2 were teenagers requesting a slimmer face, but their faces did not match either Type I or Type II criterion. Then, 39 patients (35 females and 4 males) and 22 patients (21 females and 1 male) were classified as to Type I (copious face) and Type II (ptotic lower cheeks in the aging population), respectively. The average ages in Types I and II were 43.74 ± 10.75 (19-65) and 57.09 ± 9.83 (38-76), respectively. Preoperative average volumes of each lower facial segment in Type I were as follows: The right upper (RU) left upper (LU), right lower (RD), and left lower (LD) segments were 138.89 ± 25.54 mL (99.47-199.65), 138.75 ± 22.17 mL (97.32-189.34), 110.42 ± 24.72 mL (63.11-175.37), and 110.31 ± 23.46 mL (67.95-168.02), respectively. Postoperative average volumes of each lower facial segment in Type I were as follows: RU″, LU″, RD″, and LD″ were 125.30 ± 19.32 mL (93.22-183.72), 124.10 ± 17.78 mL (88.98-174.14), 95.40 ± 19.45 mL (58.15-147.31), and 93.96 ± 18.29 mL (64.69-154.90), respectively. The preoperative average volumes of each lower facial segment in Type II were as follows: RU, LU, RD, and LD were 130.86 ± 17.01 mL (98.64-157.38), 128.29 ± 14.99 mL (104.32-156.28), 101.46 ± 23.47 mL (59.08-135.07), and 98.66 ± 22.11 mL (57.34-132.27), respectively. Postoperative average volumes of each lower facial segment in Type II were as follows: RU″, LU″, RD″, and LD″ were 124.41 ± 16.15 mL (94.34-147.64), 122.45 ± 14.29 mL (98.25-150.57), 88.17 ± 20.97 mL (50.21-125.09), and 85.04 ± 20.30 mL (51.98-120.35; Table 1), respectively. The postoperative volumetric decrease of each segment RU″-RU, LU″-LU, RD″-RD, and LD″-LD was statistically significant in both Types I and II (*P* = 0). It showed that BFP excision reduced the volume of each lower facial segment in both facial types, regardless of the resected position. However, the differences in the VRs of the bilateral upper segments (RU″-RU) and (LU″-LU) between Types I and II were statistically significant (*P* < .01). On the other hand, those in the bilateral lower segments (RD″-RD) and (LD″-LD) between Types I and II were not statistically significant. Thus, the VR of the bilateral upper facial segments (RU and LU) in Type II was less than that in Type I due to less resection of the upper position of BFP in Type II. However, there were no significant changes in the VR in the bilateral lower facial segments (RD and LD) because the same amount of BFP was resected from the lower facial segment in both types (Table 2, Figure 6).

**Table 1.** Preoperative and Postoperative Average Volume of Each Lower Facial Segment

Type	RU (mL)	LU (mL)	RD (mL)	LD (mL)	RU″ (mL)	LU″ (mL)	RD″ (mL)	LD″ (mL)
Type I	138.89 ± 25.54 (99.47-199.65)	138.75 ± 22.17 (97.32-189.34)	110.42 ± 24.72 (63.11-175.37)	110.31 ± 23.46 (67.95-168.02)	125.30 ± 19.32 (93.22-183.72)	124.10 ± 17.78 (88.98-174.14)	95.40 ± 19.45 (58.15-147.31)	93.96 ± 18.29 (64.69-154.90)
Type II	130.86 ± 17.01 (98.64-157.38)	128.29 ± 14.99 (104.32-156.28)	101.46 ± 23.47 (59.08-135.07)	98.66 ± 22.11 (57.34-132.27)	124.41 ± 16.15 (94.34-147.64)	122.45 ± 14.29 (98.25-150.57)	88.17 ± 20.97 (50.21-125.09)	85.04 ± 20.30 (51.98-120.35)

LD, preoperative average volume of the left down segment of the lower face divided into 4 parts; LD″, postoperative average volume of the left down lower facial segment; LU, preoperative average volume of the left up segment of the lower face divided into 4 parts; LU″, postoperative average volume of the left up lower facial segment; RD, preoperative average volume of the right down segment of the lower face divided into 4 parts; RD″, postoperative average volume of the right down lower facial segment; RU, preoperative average volume of the right up segment of the lower face divided into 4 parts; RU″, postoperative average volume of the right up lower facial segment.

**Table 2.** Variation Rate (VR) for Postoperative Average Volume of Each Lower Facial Segment in Types I and II

Facial segment	Type I (mL)	Type II (mL)	*P*-value
RU**″**-RU	−9.68 ± 5.27	−5.07 ± 2.50	<.01
LU**″**-LU	−10.08 ± 6.35	−4.52 ± 2.28	<.01
RD**″**-RD	−12.79 ± 9.08	−13.00 ± 5.65	.91
LD**″**-LD	−13.86 ± 8.51	−13.84 ± 6.67	.99

LD, preoperative average volume of the left down segment of the lower face divided into 4 parts; LD**″**, postoperative average volume of the left down lower facial segment; LU, preoperative average volume of the left up segment of the lower face divided into 4 parts; LU**″**, postoperative average volume of the left up lower facial segment; RD, preoperative average volume of the right down segment of the lower face divided into 4 parts; RD**″**, postoperative average volume of the right down lower facial segment; RU, preoperative average volume of the right up segment of the lower face divided into 4 parts; RU**″**, postoperative average volume of the right up lower facial segment.

**Figure 7.**
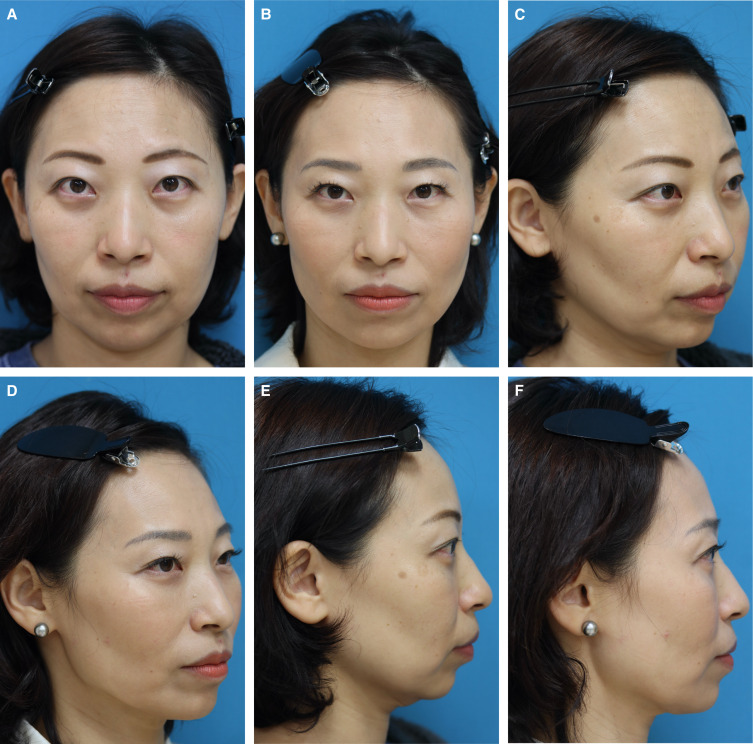
(A, C, E) A 48-year-old female presented to our clinic for treatment of a heavy lower face, which continued to worsen in recent years. This patient was categorized as Type I with severe symptoms on the left cheek, almost manifesting an additional jawline. The distal part of the buccal fat pad (BFP) extension was initially resected using the Matarasso approach, and then, gradually proceeding up to the proximal part of the BFP extension to reduce whole lower cheeks evenly. (B, D, F) Twelve-month postoperative photographs show a slimmer and lighter lower facial contouring. The last picture was taken on November 6, 2021.

**Figure 8.**
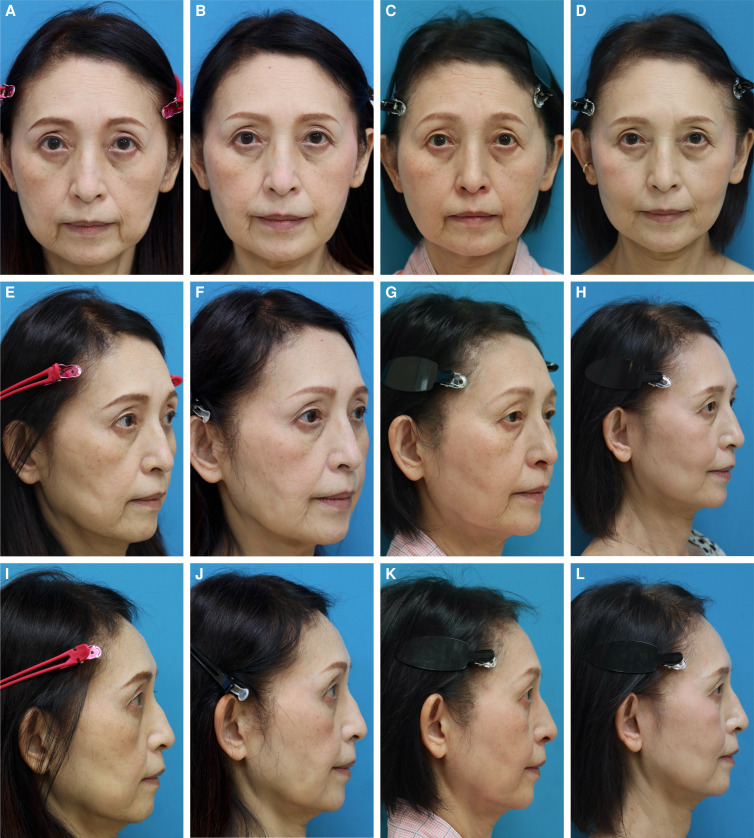
A 62-year-old female presented to our clinic seeking treatment for her lax jawline. (A, E, I) Pseudoherniation of the buccal fat pad (BFP) was observed and this patient was categorized as Type II (ptotic lower cheeks in the aging face). It was determined that the distal end of the BFP extension was the cause of the lax jawline, and on December 10, 2020, she underwent BFP excision, for which the Matrasso approach was used. (B, F, J) Twelve-month postoperative photographs show that the lower facial volume is reduced, resulting in an improvement in ptotic lower cheeks. (C, G, K) Although the patient was satisfied with the initial result, she was dissatisfied with the residual jawline as she gained body weight as shown in the photographs taken 18 months after BFP excision. She then requested further improvement with additional surgery, and a minimally invasive facelift was performed on May 18, 2022. (D, H, L) Twelve-month postoperative photographs show further improvement in the remaining jawlines. The last picture was taken on May 20, 2023.

Ninety-four patients underwent trans-conjunctival lower blepharoplasty during the period before and after or simultaneously with BFP excision, and it was the most frequent concomitant surgery. Other surgeries performed with BFP excision are presented in Table 3. There were no patients in this study who underwent facial fat injection or grafting. The satisfaction rate of the surgery was evaluated for 43 patients, who were followed up for 12 months postoperatively, by using a blind evaluator. The scale ranged from 0 to 3 (0: poor, 1: no difference, 2: better, and 3: much better). The results showed 0 for 0.8%, 1 for 3%, 2 for 57.1%, and 3 for 39.1%. None had any serious complaints. Two patients had a minor postoperative gaunt appearance (1.6%), with the symptoms of 1 patient resolving naturally 2 months postoperatively. Symptoms continued to persist in the other patient, and she confessed that she had previously undergone facial liposuction a few years prior. A long-lasting hyaluronic acid filler was injected into the inverted area of her cheek, and the symptoms significantly improved with lasting results. One female patient experienced discomfort (at a level of 0.8%) at the left upper cheek immediately after the surgery, and mild swelling of the left cheek was observed objectively. The patient stated that she developed the uncomfortable feeling when having a meal. This condition was suspected to be a minor injury to the left parotid duct. Oral nonsteroidal anti-inflammatory drug and antibiotics were administered to fully resolve symptoms without further intervention within 2 weeks postoperatively. Another female patient had a postoperative bleeding (0.8%) extending down to the left lower neck with mild swelling, although no bleeding tendencies presented in the preoperative hematological test. This resolved naturally within 4 weeks postoperatively. There were no cases of infection (0%). Possible complications associated with BFP excision and rate of occurrence in this study are presented in Table 4. Typical cases of BFP excision in a Type I face are shown in Figure 7, and 1 case of BFP excision in a Type II face, followed by a subsequent mini facelift, is shown in Figure 8.

**Table 3.** Concomitant Surgeries Performed With Buccal Fat Pad (BFP) Excision

Surgery	Previously	Simultaneously	Subsequently	Total
Trans-conjunctival lower blepharoplasty	71 (4^a^)	18	5 (2^b^)	94
Upper blepharoplasty	9	5	10	24
Facial liposuction	7 (5^a^)	2	3	12
Facelift	6 (5^a^)	1	4	11
Thread lift	5 (4^a^)	1	2	8
Facial osteotomy	4 (4^a^)	0	0	4

^a^The number of surgeries performed at other clinics. ^b^The number of revised surgeries performed at The Ginza CUVO Clinic, Tokyo, Japan.

**Table 4.** Complications and Their Rates of Occurrence

Complications	Rate of occurrence (%)
Functional	
Infection	0
Hemorrhage (hematoma)	0.8
Trismus	0
Injury to the parotid gland duct	0.8
Injury to the nerves (facial or third branch of trigeminal)	0
Appearance	
Gaunt appearance	1.6

## DISCUSSION

There is no disputing the fact that facelift is the most powerful tool for treating an aging face and has historically remained the sole and exclusive surgery. Facelift is aesthetically predictable, as it not only has a firm lift-up effect, but also erases wrinkles and lax skin in the face. The other advantages are its expandability; for example, with a better understanding of the surgical facial anatomy, it is evolving into a new dimension using not only SMAS manipulations, but also the deep plane approach, enabling the surgical range to extend to the jowl and neck. The versatility of the facelift method helps in regaining a youthful face, jowl, and neck for a wide age range of patients and various types of aging faces. Multifactorial causes attributed to aging are mentioned in the previous chapter. For instance, an aging face is facilitated by gravity force, which is partially driven by BFP weight. Therefore, BFP excision plays a role in releasing the weight burden of the lower face, making it an important option for aging face surgery as well. The northern Asian population with a relatively large BFP and good skin resilience is especially well indicated for BFP excision. The results show that BFP reduction is one of the essential factors in the facial rejuvenation process. The Matarasso approach was used in this study to grasp the far end of the distal part of BFP extension, which contributes to aging jawlines. However, the Matarasso approach is more technically demanding than other approaches due to possible encounters with abundant blood vessels and capillaries near the surrounding tissues. If not carefully dissected, spontaneous and copious bleeding may start from a small wound at the oral mucosa. Once this happens, it is not easy to secure hemostasis, because both identifying and grasping the bleeding point in the deep and narrow space inside the cheek is next to impossible. Therefore, bleeding control is one of the most important factors in BFP excision.

In order to maximize the effectiveness of this surgery, this study scrutinized the relationship between the resected position of the BFP and variations in the facial shape and volume. It is extremely important to identify all BFP-related symptoms before surgery with the method altered depending on each case, including the resection position of the BFP. For this, 61 patients were divided into 2 general types: 39 patients with a large bottom-heavy face commonly seen in the northern Asian population were categorized as Type I, and 22 patients with ptotic lower cheeks and jaw lines, often accompanied by the pseudoherniation of BFP, were classified as Type II. Interestingly, the average age of Type II patients was 13.4 years older than that of Type I, validating the presence of ptotic lower cheeks later in life. Facial photographs were taken from multiple angles using 3D photography, and these enabled a measurement of the lower facial volume to determine the postoperative changes. An analysis of these data revealed a correlation between the resected position of the BFP and its influence on facial volume.

It was found that the lower facial volume of Type I patients was greater than that of Type II patients (Table 1). This was attributed not only to the large and heavy or bottom-heavy face, but also to the large “chipmunk face” of younger patients also included in Type I. In contrast, Type II patients were older, and their cheeks were more deflated than Type I patients. Therefore, the lower facial volume in Type II was smaller than that in Type I. Although BFP extension in Type II was droopier and more descended, the volume of the upper segment (RU and LU) in the lower face was still greater than that of the lower segment (RD and LD; Table 1). This is attributed to the fact that the volume measured by 3D photo analysis includes not only the BFP, but also superficial fat layers, salivary glands, mimetic muscles, and bones. It is presumed that the volumetric effect of the facial components on the upper segments is more than the effect of the BFP descent on the lower segments.

In Type I patients, the distal part of the BFP extension was first resected, extending to the proximal part to reduce the whole lower cheek. Their resilient and thick skin would mostly compensate for the reduced cheek after complete resection. In Type II patients, the distal part of the BFP extension is responsible for the ptotic lower cheeks or pseudoherniation. Therefore, priority should be given to resect only the ptotic region or herniated (distal) part of the BFP, as shown in Figure 1. This surgery should be performed carefully in Type II patients to avoid over resection, which may cause the postoperative appearance. Because the upper segment of the lower cheek in Type II patients is frequently deflated, a further resection of the BFP in this part may worsen symptoms. To summarize the technical challenges involved in Type I patients compared with Type II, the BFP of Type I patients can be removed by ignoring any negative outcome if it is resected evenly from the whole BFP extension, while only the distal end of the BFP extension should be gradually removed in Type II patients so that there is no negative impact.

This study revealed that the section of the BFP extension resected in Type I patients significantly altered both the upper and the lower facial shape and volume, with a major part of the BFP extension from the lower face resected. The data show that the shape and volume of only the lower facial segment in Type II patients decreased with only the distal part of the BFP extension resected (Table 1, Figure 6). These findings imply that the outcomes of BFP excision are more precise when the resected position and amount of BFP are varied depending on face type. When the relationship between the resected position and its influence on facial shape and volume is well understood, BFP excision can become a better tool for facial rejuvenation. However, a limitation of this study was that some patients presented with symptoms classified as both facial types. Therefore, this study provides a small reference to the resected BFP position and its influence on the facial shape and volume.

**Figure 9.**
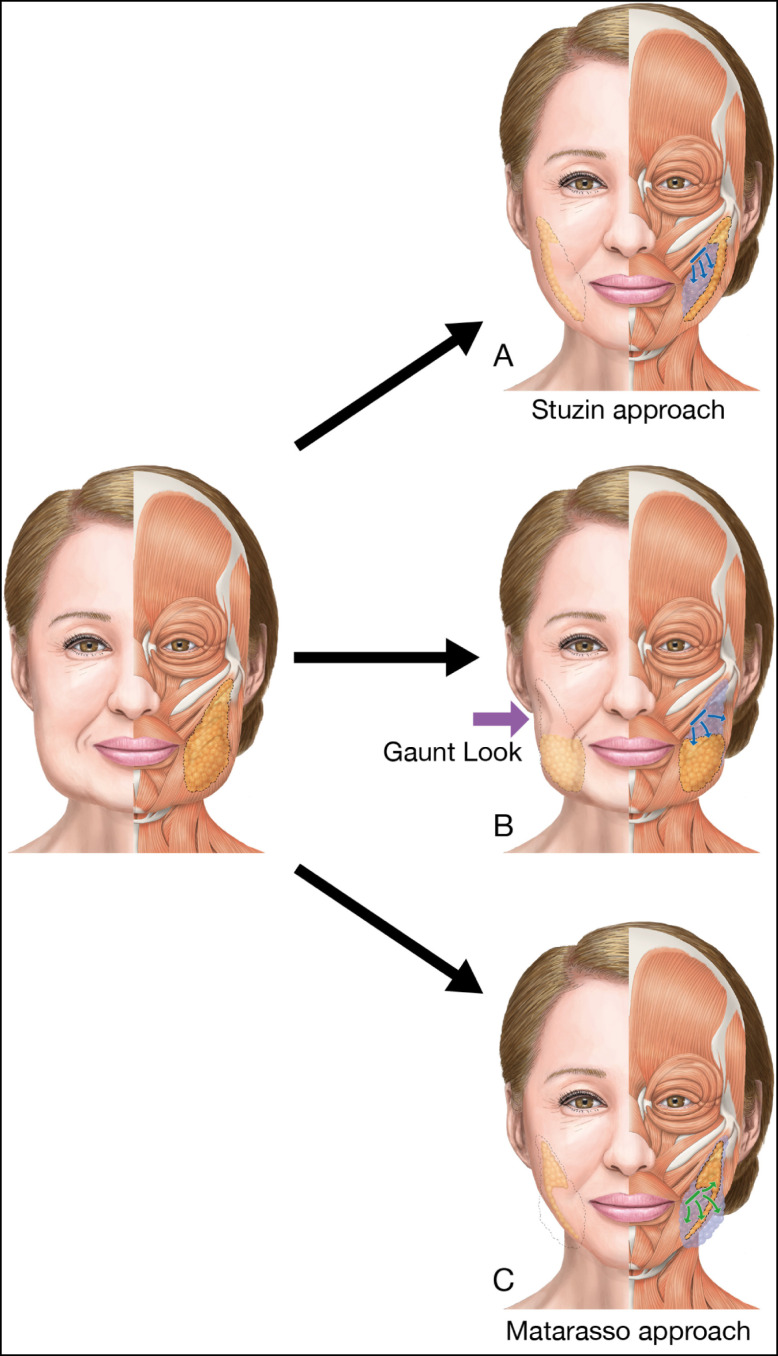
The illustration on the left shows a typical aging face with ptotic lower cheeks. The left half of the face is pictured as normal with skin coverage, while the right half is schematic without skin to show the facial muscles and buccal fat pad (BFP) responsible for the typical aging jawlines. (A) The resection of the proximal BFP extension using the Stuzin approach (indicated by small arrows) is shown. The resection should be continued down to the distal BFP extension to have a smooth contour of the face. (B) A pitfall with the proximal part of the BFP extension over resected using the Stuzin approach is shown, while the distal part of the BFP extension remains. The gap between the vacant upper cheeks and the remaining BFP extension will exaggerate the postoperative gaunt appearance (an arrow) because of its location below the zygoma, which is responsible for cheek fullness. (C) A gradual excision of the BFP extension starting from its distal end by the Matarasso approach (indicated by small arrows) is shown, and it will lead to a smoother facial contour with a reduced jawline.

Although only a few minor postoperative complications were encountered in this study, there are other possible complications associated with this surgery. First, the postoperative gaunt appearance (hollowness of the cheeks) is caused by an over resection of the BFP at the upper cheek.^17^ Many patients show some reluctance to undergo BFP excision because of this risk, which has led to a stigmatization of this surgery. This occurs most commonly in the aging face when a trans-section is made at the pseudoherniated BFP enveloping the fascia near the facial surface.^18^ Even a minimal over resection of the BFP near the facial surface in an aging face can have a negative impact on facial contours. However, it is less likely to occur in a large face due to the buccal lipodystrophy associated with youthfulness.^19^ When the BFP in an aging face is resected using the Stuzin approach, the chances of occurrence of the postoperative gaunt appearance are slightly higher. Because of its location just below the zygoma, the proximal part of the BFP extension plays an important role in cheek fullness^20^ and is easily grasped with this approach, with the upper portion of the cheeks principally reduced at the start of the procedure, as shown in Figure 9A. Over resection of this area, especially if the distal part of the BFP extension remains intact, and discrepancies between a resected and a nonresected BFP might exaggerate the postoperative gaunt appearance, as shown in Figure 9B. Alternatively, the Matarasso approach primarily enables the resection of the distal part of the BFP extension with the lower cheek mainly reduced, as shown in Figure 9C. Therefore, the postoperative gaunt appearance is less likely to occur with this approach for the treatment of ptotic lower cheeks.

It is worth mentioning that knowing the exact cessation timing of the BFP excision is also critical to avoid over resection and the postoperative gaunt appearance. A clue for termination is to feel a retraction force driven from the remaining BFP, which tends to withdraw backward into the BFP pocket when it is pulled with forceps. This is due to the remaining BFP being attached to the surrounding tissue by retaining ligaments. On the other hand, if its retraction force is still weak and the remaining BFP does not fully withdraw into the BFP pocket, it should be resected further until the remaining BFP is automatically hidden into the pocket. When this retraction force becomes stronger as the resection proceeds and it starts snapping back, it is a clear sign of termination.

The postoperative gaunt appearance may also be caused by rough dissection ignoring surgical planes, potentially damaging delicate tissues around deeper cheek regions. Damage to the vascular pedicles might induce profuse bleeding or severe hematoma as a result. Tissue damage from hematoma will not only impair the normal healing process but can also facilitate subcutaneous tissue scarring. This may affect the facial surface, leading to a contour deformity or inversion of the cheek surface, mimicking the postoperative gaunt appearance. Hence, BFP excision should be always carried out carefully following the surgical plane to restore anatomical structures as much as possible.

Injury to the nerves and the parotid duct, infection, hematoma, and trismus caused by massive swelling are the other functional complications associated with BFP excision.^21^ Among these, bleeding followed by hematoma is the most frequently encountered complication. It is scientifically proven that cortisol hormones in the blood increases with anxiety and stress from surgery,^22^ facilitating inflammation and pain over time. Also, hypertension caused by surgical overstrain may result in increased intraoperative and postoperative bleeding, negatively impacting postoperative recovery. Hence, intravenous sedation should always be administered prior to BFP excision. When bleeding is mild to moderate, hematoma can often dissolve within a few weeks without intervention. However, if bleeding is severe, especially due to injury to the facial vein running laterally to the BFP, it can result in massive bleeding, followed by chronic hematoma, which may take one full month to heal completely. Prolonged hematoma may cause facial contour deformity as a late-stage complication. Injury to the facial nerves and mandibular branch of the trigeminal nerves might also be caused by blind dissection and by ignoring the planes of nerves located within reachable distance by surgical tools during the dissection. Nerve injuries should be completely avoided as mechanical damage to the proximal trunk deep inside the face could be irreversible.

**Figure 10.**
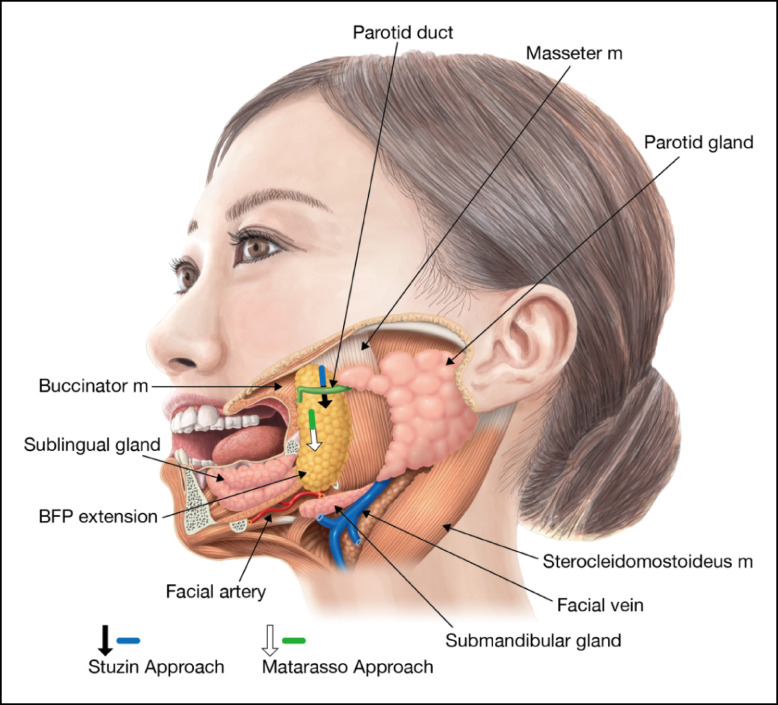
If the Matarasso approach is chosen (the line below the parotid gland duct), the entry point is made approximately 1 cm inferior to the parotid duct on the oral vestibule with dissection starting distal and proceeding posterior-laterally and posterior-inferiorly. Noticeably, dissection with this approach moves away from the parotid duct (shown as the arrow), greatly reducing the risk of injury to the parotid duct. If the Stuzin approach is chosen (the line above the parotid gland duct), the entry point is made at the oral sulcus above the parotid duct. This dissection proceeds posterior-inferiorly passing under the parotid duct (shown as the arrow). Therefore, injury may occur if dissection around the parotid duct is not made carefully.

Injury to the parotid gland (Stensen duct) is another significant complication associated with this surgery, and serious swelling may occur because of an obstruction in saliva secretion. This may heal if the injury is mild, but if it is severe, evacuation measures should be undertaken, and on rare occasions, an ENT doctor should be consulted. As discussed in the previous section, the distal part of the BFP extension responsible for the ptotic jawline should be resected as a matter of priority in an aging face. If the Matarasso approach is chosen, the entry point is made approximately 1 cm inferior to the parotid duct on the oral vestibule so that dissection begins posterior–laterally and inferiorly to the BFP pocket. Since this direction leads away from the parotid duct, injury to the parotid duct is extremely rare. On the other hand, if the Stuzin approach is used, an entry point is made at the oral sulcus above the parotid duct and its dissection proceeds posterior-inferiorly under the parotid duct. Injury may occur to the parotid duct if the dissection is rough and careless while overlooking the parotid duct. The relationship between the parotid duct and the respective surgical approaches in BFP excision in the aging face is shown in Figure 10.

Although BFP excision is relatively indicative for northern Asian ethnicities whose skin is thick and resilient, its mere excision does not necessarily improve droopy jawlines completely. In such cases, subsequent facelift procedures should be considered to resolve remaining symptoms. One relapsed case was presented in this study after the BFP excision (Figure 8). The patient's skin was relatively thin and attenuated compared with that of a typical northern Asian. She had some amount of pseudoherniated BFP, making her jowl look more attenuated and saggier. A diagnosis of the pseudoherniated BFP was made in her case when the reproducibility of the BFP was confirmed by using an external force. Thus, BFP excision was performed, which initially produced fair results 12 months postoperatively. But some of the jawline issues remained and there was continued relapse as she gained weight during the pandemic period. The cause of this relapsing jawline was considered to be skin loosening and a descending superficial jowl fat, which was attributed to volume increase with her weight gain. Eighteen months after the initial BFP excision, a subsequent mini facelift was performed with additional liposuction to improve the jawlines. The result gained 12 months after the mini facelift showed further improvement. This demonstrated that the simple mini facelift worked well in collaboration with the previous BFP excision, after the weight burden of the BFP from her lower face was reduced. This case would otherwise have been indicative of a full facelift.

If simple subcutaneous dissection and SMAS plication within a limited cheek area were to be performed in a mini facelift as the first surgery for a northern Asian patient with a large BFP, it would not be able to resolve the jowl symptom completely, as the effect of the facelift might relapse with time due to the gravity force driven by the BFP. As evidence of this phenomenon, the author has occasionally encountered patients seeking resolution for relapsed jawlines after a mini facelift surgery. If the untouched BFP was identified as a culprit of this symptom, subsequent BFP excision was carried out with favorable results in most patients. As can be seen in this case, a combination of BFP excision and mini facelift procedures was helpful in restoring a youthful appearance. In fact, previous papers suggest that an external approach to BFP excision during facelift has excellent results while avoiding intraoral incision and unnecessary contamination.^23^ Therefore, it is worth considering the intraoperative BFP excision during facelift in the case of patients with a recognizably sized BFP. The previous paper also discussed the safety of excising BFP by the external approach during the facelift, when utilizing measurements from facial landmarks as a guide to identify the BFP extension and to avoid injuries to the surrounding tissues.^24^ However, the surgeon must still pay great attention to avoid injuries to the facial nerves, parotid duct, and vascular pedicle. Above all, facial nerves have many variant pathways near the BFP extension, and there is always a risk of facial nerve injury.^25^ Hence, BFP excision using the intraoral approach has been utilized as a priority, although the external approach during facelift is ideal, which help avoid performing separate surgeries at different times.

To improve the accuracy of the surgical result, precise preoperative examinations are imperative before considering the specific surgery for any aging face. For instance, a Caucasian skin is relatively thin and delicate, while the skin of northern Asians is typically thick and strong.^26^ Therefore, it is not uncommon that facelift is given priority among the Caucasian population to resolve wrinkles and loose skin around jowls. On the other hand, a northern Asian face is more tolerant to wrinkles and loosening of skin. Therefore, the total number of facelifts performed on Asian patients is far less than that of Caucasians.^27^ On the other hand, the amount of BFP seems to be less in Caucasians and greater in northern Asians, therefore, suggesting that in this demographic, BFP excision could become as popular as facelift.

In some aging patients with reduced volume in the midface presenting as deflated cheeks, surgeons may choose to reposition the descended BFP to its original position at the upper cheek with SMAS tightening or to perform a fat graft during a facelift to increase the volume. It is important to note the risk of a negative outcome if BFP excision is performed on this type of aging face. BFP excision is contraindicated for deflated types of aging faces with atrophic BFP, which is frequently seen in an aging Caucasian population. It is, therefore, vital to understand the fundamentally different purposes of facelift surgery and BFP excision. The former is performed for whole facial rejuvenation, namely, for improving facial wrinkles as well as facial redundancy and jawlines, and the latter is indicated selectively for minor ptotic lower cheeks, which were anatomically recognized as the descent of the BFP into the premasseteric space.^23^ BFP excision is not a substitute for facelift, nor can BFP excision yield precise results. However, it is possible for BFP excision to complement the effects of a facelift by reducing the weight and volumetric burden of the BFP.

Therefore, a careful observation of the jawlines and estimation of cheek and BFP volume must be considered prior to deciding whether adding mid-face volume or reducing lower face volume is appropriate. Preferred conditions for either BFP excision or facelift in an aging face are concisely shown in Table 5. Even so, there is always a risk of skin loosening after BFP excision regardless of ethnicity. In such cases, it is better to allow a natural restoration with adjunct skin care and recommend a proper facelift if symptoms persist. But, in the case of an aging patient with a severely attenuated skin, especially in Caucasians, BFP excision is contraindicated in the first place regardless of the size of the BFP, since there is a high risk of marked skin loosening around jowls. In response to this kind of severely aging face, more advanced state-of-the art–type facelifts may be considered, such as “an extended deep plane facelift”, which may resolve all symptoms in only 1 surgery.^28^

**Table 5.** Preferred Conditions for Surgery

Surgery	Preferred conditions
BFP excision	Mild-to-moderate aging face with large and heavy BFP Middle aged or selected elderly patients Thick and good resilient skin type with no deflated cheeks Northern Asians
Facelift	Moderate-to-severe aging face with excess skin and wrinkles Middle aged to elderly patients Thin and attenuated skin type with deflated cheeks Caucasians

Although indications for BFP excision are similar to that of facelift surgery, there are preferred patient selection conditions for both surgeries. BFP, buccal fat pad.

## CONCLUSIONS

Facelifts have been a dedicated treatment for aging faces throughout history. Recently though, BFP excision has proved effective in appropriate candidates with a large BFP causing sagging jawlines. The associated risks of BFP excision are reduced when surgical position and influence on the facial shape and volume are thoroughly investigated. In many cases, subsequent facelifts may reinforce BFP excision to produce a further marked effect. It is always better to perform a careful examination when selecting appropriate candidates for different facial rejuvenation surgeries.

## Supplementary Material

ojad089_Supplementary_Data
